# Functional Scanning of Apple Geminivirus Proteins as Symptom Determinants and Suppressors of Posttranscriptional Gene Silencing

**DOI:** 10.3390/v10090488

**Published:** 2018-09-11

**Authors:** Binhui Zhan, Wenyang Zhao, Shifang Li, Xiuling Yang, Xueping Zhou

**Affiliations:** 1 State Key Laboratory for Plant Disease and Insect Pest, Institute of Plant protection, China Academy of Agricultural Sciences, Beijing 100193, China; binhuizhan@126.com (B.Z.); 13764672176@163.com (W.Z.); 2 State Key Laboratory for Rice Biology, Institute of Biotechnology, Zhejiang University, Hangzhou 310058, China

**Keywords:** Apple geminivirus, RNA silencing suppressor, symptom determinant

## Abstract

Apple geminivirus (AGV) is a recently identified geminivirus which is isolated from the apple tree in China. We carried out functional scanning of apple geminivirus proteins as symptom determinants and suppressors of posttranscriptional gene silencing (PTGS). Our results indicated that AGV V2 is an important virulence factor localized to the nucleus and cytoplasm that suppresses PTGS and induces severe symptoms of crinkling and necrosis. AGV C1 is also a virulence determinant which elicits systemic necrosis when expressed from a PVX-based vector. The AGV C4 is targeted to cytoplasm, plasma membrane, nucleus, and chloroplasts. The inoculation of PVX-C4 on *N. benthamiana* induced severe upward leaf curling, which implied that AGV C4 also functions as a symptom determinant, and mutation analyses suggested that the acylated residues on Gly2 and Cys8 play important roles in its subcellular localization and symptom development.

## 1. Introduction

Geminiviruses are a large family of single-stranded DNA viruses that can infect a broad range of plants in most parts of the world [1,2]. According to the taxonomic criteria, the family *Geminiviridae* is divided into nine genera: *Becurtovirus*, *Begomovirus*, *Curtovirus*, *Eragrovirus*, *Mastrevirus*, *Topocuvirus*, *Turncurtovirus*, *Capulavirus*, and *Grablovirus* [1,3]. Over the last decade, the development of new molecular techniques and bioinformatics has greatly accelerated the discovery of novel geminiviruses, and 12 geminiviruses have been reported to infect woody plants recently. Among them seven geminiviruses infecting jatropha belong to the genus *Begomovirus* [4,5,6], grapevine red blotch virus belongs to the genus *Grablovirus* [7,8,9], while citrus chlorotic dwarf associated virus [10], mulberry mosaic dwarf associated virus [11], apple geminivirus (AGV) [12], and grapevine geminivirus A [13] need taxonomic assignment. 

AGV was identified in 2015 from an apple tree in China, and it contains the typical geminivirus-like nanonucleotide motif TAATATTAC and displays a distinctly geminivirus-like genomic organization [12]. The AGV genome consists of a single ~2.9 k nucleotides circular ssDNA that contains six open reading frames (ORFs) on both strands of the double-stranded replicative form DNA. The sense strand encodes ORF V1 and ORF V2, and the complementary strand encodes C1, C2, C3, and C4 [12]. In this study, we have systematically scanned the functions of AGV-encoded proteins in symptom development and silencing suppression, and found that AGV V2 is an important virulence determinant which can suppress posttranscriptional gene silencing (PTGS) and induce severe crinkling and necrosis; AGV C1 is also an important virulence determinant which elicits systemic necrosis when expressed from a PVX-based vector; AGV C4 functions as a symptom determinant, and the putative acylated residues Gly2 and Cys8 play important roles in its subcellular localization and symptom development.

## 2. Materials and Methods

### 2.1. Construction of Plasmids

To construct binary plasmids transiently expressing GFP fusion proteins for subcellular localization assays in *Nicotiana benthamiana*, the coding sequences of AGV V1, V2, C1, C2, C3, and C4 were amplified with the corresponding primer pairs, digested with X*ba*I and S*ma*I restriction enzymes and inserted into the binary vector pCam35S-GFP to generate the recombinant vectors pCam35S-V1-GFP, pCam35S-V2-GFP, pCam35S-C1-GFP, pCam35S-C2-GFP, pCam35S-C3-GFP, and pCam35S-C4-GFP expressing the fusion proteins V1-GFP, V2-GFP, C1-GFP, C2-GFP, C3-GFP, and C4-GFP.

To construct binary plasmids transiently expressing proteins for PTGS assays, the coding sequences of AGV V1, V2, C1, C2, C3, and C4 were amplified and cloned into the restriction sites on the binary vector pCHF to obtain pCHF-V1, pCHF-V2, pCHF-C1, pCHF-C2, pCHF-C3, and pCHF-C4 recombinant plasmids for agroinfiltration.

For PVX assays, the full-length coding sequence of AGV V1, V2, C1, C2, C3, and C4 were PCR-amplified with the primer pairs listed in Table S1. The products were digested with the corresponding restriction enzymes and ligated to the pGR106 vector to generate PVX-V1, PVX-V2, PVX-C1, PVX-C2, PVX-C3, and PVX-C4.

The mutants C4_G2A_, C4_C8A_, and C4_G2AC8A_ were constructed using Fast Mutagenesis System (Transgen, Beijing, China) with primers covering and flanking the mutation sites. To analyze the subcellular localization of the C4 mutants, the coding sequences of C4_G2A_, C4_C8A_, and C4_G2AC8A_ were amplified using the primer pairs listed in Table S1, digested with the corresponding restriction enzymes and inserted into the expression vector pCam35S-GFP. The recombinant vectors pCam35S-C4_G2A_-GFP, pCam35S-C4_C8A_-GFP, and pCam35S-C4_G2AC8A_-GFP were designed to express fusion proteins C4_G2A_-GFP, C4_C8A_-GFP, and C4_G2AC8A_-GFP. To investigate the pathogenicity of the C4 mutants, the coding sequences of C4_G2A_, C4_C8A_, and C4_G2AC8A_ were amplified and cloned into pGR106 resulting in PVX-C4_G2A_, PVX-C4_C8A_, and PVX-C4_G2AC8A_, respectively.

All primers and restriction enzyme sites used for plasmid construction are listed in supplemental Table S1.

All constructs were sequenced before use to make sure no amplification errors were induced by PCR.

### 2.2. Agroinfiltration

The recombinant PVX infectious clones were transformed into *Agrobacterium tumefaciens* strain GV3101 and the binary expression vectors were transformed into EHA105. After incubating in Luria–Bertani broth with the appropriate antibiotics at 28°C overnight, the cultures were centrifuged and resuspended with infiltration buffer (10 mM MES, pH 5.7, 10 mM MgCl_2_, 150 mM acetosyringone) to a final OD_600_ of 0.5–1.0. After incubating at room temperature for 3 h, the suspensions were infiltrated into leaves of 4-week-old *N. benthamiana* plants. The infiltrated plants were analyzed for fluorescence images by confocal laser scanning microscopy (CLSM) at 3 d post infiltration.

### 2.3. Fluorescence Observation

To investigate the subcellular localization of GFP fusion proteins, fluorescence images of the epidermal cells of *N. benthamiana* infiltrated with the transformed agrobacterium were captured with a Zeiss LSM 880 CLSM using the preset settings for GFP (with 488 nm excitation and 500–550 nm emission) and for chloroplast autofluorescence (with 561 nm excitation and 650–750 nm emission).

### 2.4. H_2_O_2_ Detection in Plants

Hydrogen peroxide detection in leaves of *N. benthamiana* was conducted with the 3, 3’-diaminobenzidine (DAB)-HCl uptake method as described previously with minor modifications [14]. Briefly, the leaves were cut off at the base of the stem and then immersed in 1mg/mL DAB solution in Tris-HCl buffer (pH 3.8). After 12 h incubation at room temperature, the leaves were bleached with 96% ethanol in boiling water for 5 minutes and photographed. This method was used to decolorize the leaves and detect the H_2_O_2_ content with the dark brown precipitation produced by the reaction of DAB with H_2_O_2_.

### 2.5. PTGS Assay

Agrobacterium cultures carrying 35S-GFP, 35S-P19, pCHF-V1, pCHF-V2, pCHF-C1, pCHF-C2, pCHF-C3, and pCHF-C4 were prepared as described before with a final OD_600_ of 1.0. The pCHF3 + GFP agrobacterium culture mixture (harboring pCHF3 and 35S-GFP), V1 + GFP agrobacterium culture mixture (harboring pCHF3-V1 and 35S-GFP), V2 + GFP agrobacterium culture mixture (harboring pCHF3-V2 and 35S-GFP), C1 + GFP agrobacterium culture mixture (harboring pCHF3-C1 and 35S-GFP), C2 + GFP agrobacterium culture mixture (harboring pCHF3-C2 and 35S-GFP), C3 + GFP agrobacterium culture mixture (harboring pCHF3-C3 and 35S-GFP), C4 + GFP agrobacterium culture mixture (harboring pCHF3-C4 and 35S-GFP), and P19 + GFP agrobacterium culture mixture (harboring 35S-P19 and 35S-GFP) were infiltrated into the seedlings of *GFP*-transgenic *N. benthamiana* plant line 16c. GFP fluorescence was detected under a hand-held 100W, long-wave UV lamp (UV products, Upland, CA, USA). The seedlings were photographed with a Nikon 80D digital camera (Nikon, Tokyo, Japan) with a yellow filter.

### 2.6. RNA and Protein Analyses

Total RNA was extracted from virus-infected leaves with TRIzol reagent (Invitrogen, Carlsbad, CA, USA). For northern blotting analysis of PVX RNA, 5 μg of total RNA from each sample were separated on a 1.5% denaturing agarose gel, transferred to a Hybond-N^+^ membrane (GE Healthcare, Piscataway, NJ, USA), hybridized and detected with the DIG High Prime DNA Labeling and Detection Starter Kit II (Roche Diagnostics, Mannheim, Germany). The probe was synthesized with the PCR DIG Probe Synthesis Kit (Roche Diagnostics, Mannheim, Germany) with the primers listed in Table S1.

Total proteins were extracted with extraction buffer (50 mM Tris-HCl, pH 7.5, 150 mM NaCl, 3 mM MgCl_2_, 1 mM EDTA, 1 mM DTT) containing protease inhibitor cocktail (Roche Diagnostics, Mannheim, Germany), which was followed by centrifugation at 3000 *g* for 20 min. The resulting supernatant was used as total proteins. The nuclear and cytoplasmic proteins were extracted with the Plant Nuclear and Cytoplasmic Proteins Extraction Kit (BestBio, Shanghai, China) and membrane proteins were extracted with Plant Membrane Proteins Extraction Kit (BestBio, Shanghai, China) from plant tissues. Western blot was conducted with the anti-GFP and anti-mCherry mouse monoclonal antibodies (Transgen, Beijing, China).

## 3. Results

### 3.1. Subcellular Localization

**Figure 1:**
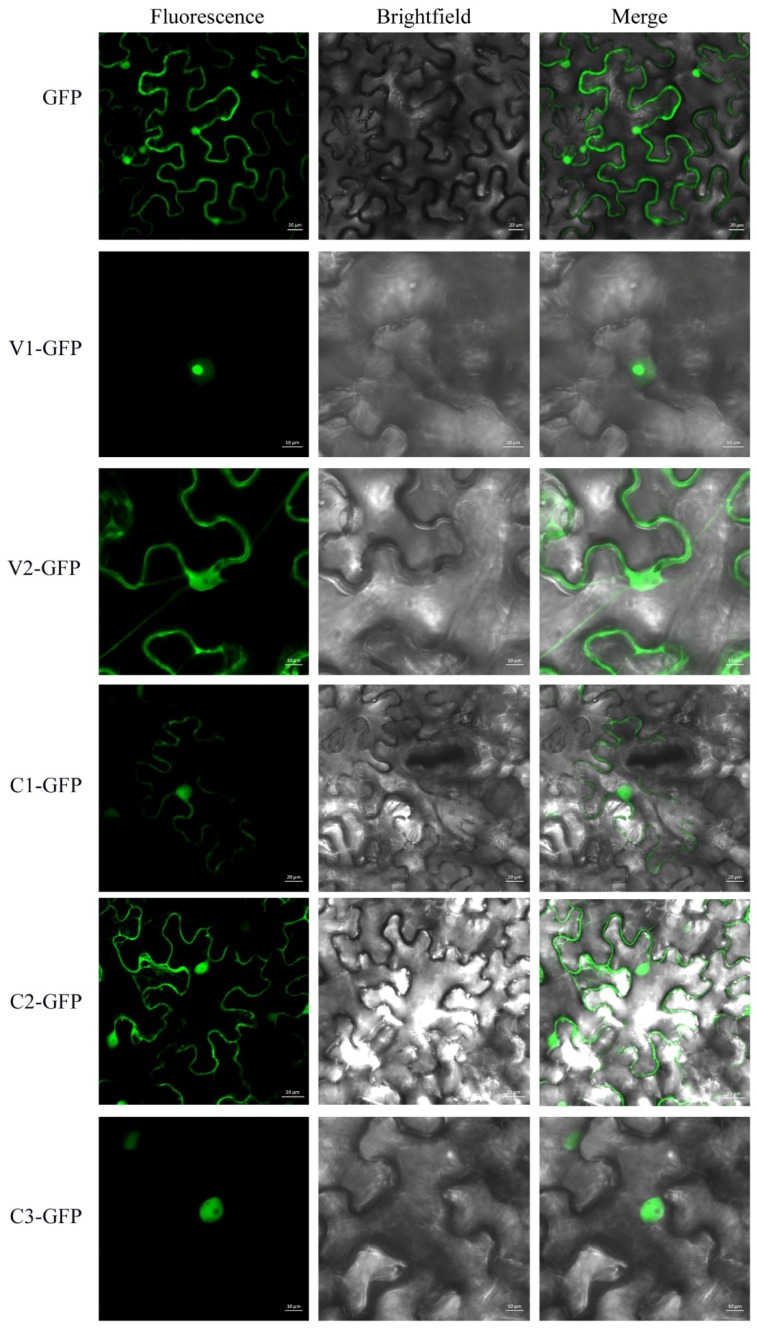
Subcellular localization of AGV V1, V2, C1, C2, and C3 in *N. benthamiana* leaves. The GFP expressed from pCam35S-GFP is distributed evenly in the cytoplasm and nucleus. The V1-GFP fusion protein from pCam35S-V1-GFP is localized to the nucleus especially the nucleolus in the infiltrated leaves of *N. benthamiana*. The V2-GFP from pCam35S-V2-GFP and C1-GFP from pCam35S-C1-GFP are distributed in the cell cytoplasm and nucleus, without nucleolus accumulation. The localization of C2-GFP from pCam35S-C2-GFP is mainly in the nucleus with bits of fluorescence in the cytoplasm. The subcellular localization of C3-GFP from pCam35S-C3-GFP is targeted to the nucleus but not to the nucleolus. Confocal laser scanning images were taken approximately 3 d post infiltration. Scale bars indicated in the photos represent 10 or 20 μm.

**Figure 2:**
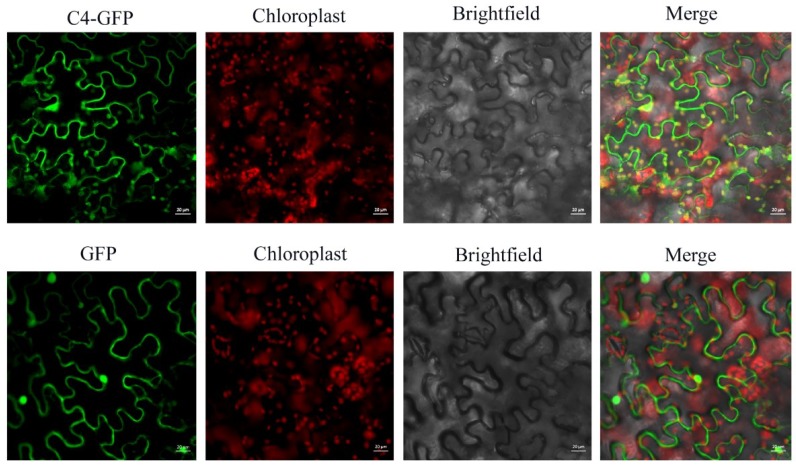
Subcellular localization of AGV C4 in *N. benthamiana* leaves. The recombinant fluorescent protein C4-GFP expressed from pCam35S-C4-GFP is localized to cytoplasm, plasma membrane, nucleus, and chloroplasts, which is different from the free GFP from pCam35S–GFP distributed evenly in the cytoplasm and nucleus. Chloroplasts are identified by the auto-fluorescence of chlorophyll through laser excitation of 561 nm. Confocal laser scanning images were taken approximately 3 d post infiltration. Scale bars indicated in the photos represent 20 μm.

Determination of the subcellular localization of a viral protein is one of the crucial steps to unravel its putative functions during virus infection. To understand the basic characteristics of AGV proteins, subcellular localization of AGV encoded V1, V2, C1, C2, C3, and C4 was investigated using GFP tag after agroinfiltration in epidermal cells of *N. benthamiana*. The full-length sequences were inserted at the N-terminus of GFP in the vector pCam35S-GFP, obtaining fusion proteins V1-GFP, V2-GFP, C1-GFP, C2-GFP, C3-GFP, and C4-GFP, respectively. The vector pCam35S-GFP expressing free GFP was used as control. The agroinfiltrated leaves were examined by CLSM at 3 d post infiltration and results are shown in Figure 1 and Figure 2. As expected, free GFP was observed in the cytoplasm and nucleus almost uniformly. The fusion V1-GFP accumulated exclusively in the nucleus, especially in the nucleolus. V2-GFP and C1-GFP were distributed in the cytoplasm and nucleus, without nucleolus accumulation. The fluorescence of C2-GFP shows nuclear-cytoplasmic distribution, with higher relative concentration in the nucleus than free GFP. C3-GFP was targeted to the nucleus but not to the nucleolus. The fluorescence of C4-GFP was detected in the cytoplasm, plasma membrane, nucleus, and chloroplasts (Figure 2). The localization in chloroplasts was indicated by the overlap with the auto-fluorescence of chlorophyll. The subcellular localization of C4 was also confirmed through subcellular fraction assays. The total proteins, nuclear proteins, cytoplasmic proteins, and membrane proteins were extracted from plant tissues expressing C4-GFP, GFP, and PIP2A-mCherry. The GFP acted as markers of nuclear and cytoplasmic proteins, while PIP2A-mCherry acted as a membrane-localization marker. Western blot analysis further indicated that C4-GFP was present in the cytoplasm, nucleus, and membrane fraction (Figure S1).

### 3.2. AGV V2 is a Suppressor of PTGS

**Figure 3:**
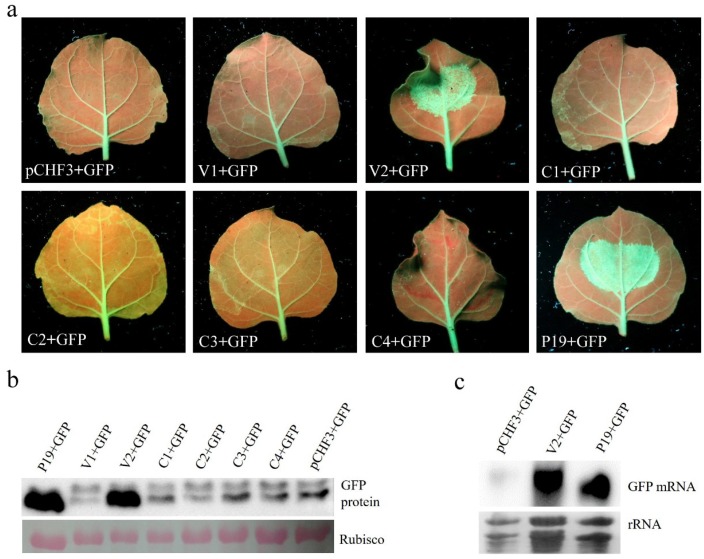
Suppression of local PTGS by AGV V2. (a) The leaves of *GFP*-transgenic 16c line were co-infiltrated with agrobacterium suspension harboring 35S-GFP expressing GFP and one of the recombinant vectors expressing AGV proteins as indicated below the images. The leaves expressing pCHF3 + GFP were used as negative control and leaves expressing P19 + GFP were used as positive control. The photographs were taken under UV light at 5 d post infiltration. (**b**) Western blot analysis of GFP accumulation in the co-infiltrated leaf patches at 5 d post infiltration. The Ponceau-stained rubisco indicates the equal loading of total proteins. (**c**) Northern blotting analysis of GFP mRNA accumulation from the co-infiltrated leaf patches at 5 d post infiltration. The rRNAs below indicate the equal loading of total RNAs.

Recently, V2 (AV2), C2, and C4 of several geminivirus species have been shown to be RNA silencing suppressors (RSSs) of PTGS, blocking local or systemic RNA silencing. To identify which viral protein(s) can suppress PTGS in AGV, we used co-infiltration assays in GFP-transgenic *N. benthamiana* 16c plants. The Agrobacterium cultures harboring one of the constructs capable of expressing AGV proteins or GFP from the CaMV 35S promoter (V1 + GFP, V2 + GFP, C1 + GFP, C2 + GFP, C3 + GFP, and C4 + GFP) were infiltrated into 16c plants. The pCHF3 + GFP Agrobacterium culture harboring empty vector was infiltrated into 16c plants as negative control and P19+GFP Agrobacterium culture expressing the P19 silencing suppressor of tomato bushy stunt virus was infiltrated as positive control. At 5 d post infiltration, the intensity of green fluorescence in the leaves expressing C1 + GFP, C2 + GFP, C3 + GFP, C4 + GFP, and V1 + GFP declined substantially, similarly to what is observed in the leaf infiltrated with the empty vector pCHF3 + GFP. In contrast, the leaf expressing V2 + GFP showed obvious and stronger green fluorescence under UV light similar to that produced by P19 + GFP (Figure 3a), which correlates with the enhanced accumulation of GFP proteins by Western blot analysis (Figure 3b). Northern blotting also showed that the concentration of GFP mRNA was significantly higher in the leaf patches co-infiltrated with V2 + GFP or P19 + GFP than in those co-infiltrated with pCHF3 + GFP (Figure 3c). These results suggest that AGV V2 is an efficient and strong RSS.

### 3.3. V2 and C1 Potentially Enhance the Pathogenicity of PVX and C4 is a Putative Symptom Determinant

**Figure 4:**
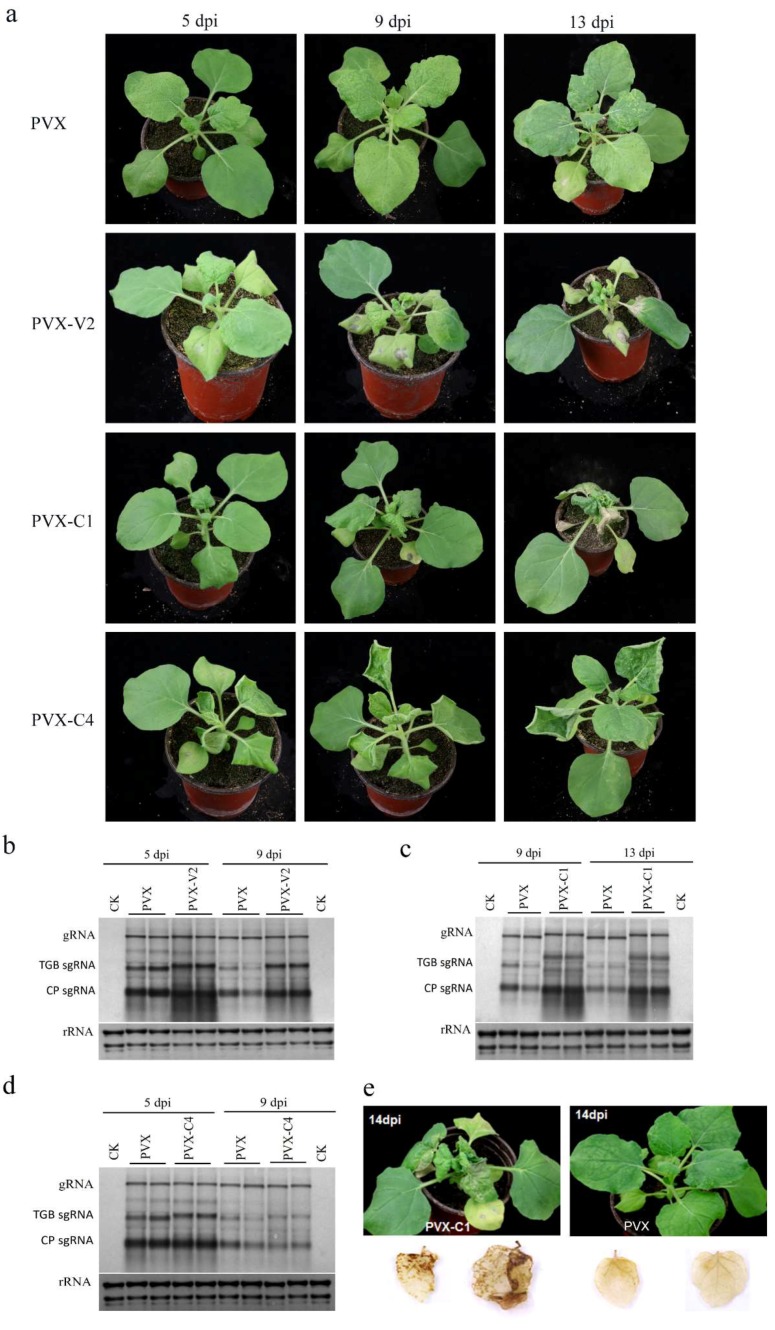
AGV V2 and C1 enhance pathogenicity of chimeric PVX and AGV C4 is a putative symptom determinant. (**a**) Symptoms of *N. benthamiana* plants inoculated with PVX, PVX-V2, PVX-C1 and PVX-C4. Symptoms were photographed at 5 dpi, 9 dpi, and 13 dpi. The images shown are representative plants out of six plants with each construct in at least three independent experiments. Northern blotting analyses of PVX-V2 (**b**), PVX-C1 (**c**), and PVX-C4 (**d**) accumulation compared with PVX. The blot shown is a representative result of three independent experiments. The probe used was specific for PVX RNA. (**e**) DAB staining for detection of H_2_O_2_ accumulation in PVX-C1- and PVX-inoculated *N. benthamiana* plants at 14 dpi, showing the higher accumulation of H_2_O_2_ in PVX-C1 inoculated leaves.

To investigate the pathogenicity of AGV proteins, the six AGV proteins were expressed from a PVX vector. The full-length *V1*, *V2*, *C1*, *C2*, *C3*, and *C4* genes were cloned into a PVX vector and inoculated into *N. benthamiana* by agroinfiltration. The inoculated plants were maintained in a growth room (24 °C day/22 °C night, 16 h light/8 h dark) and symptoms were recorded periodically. Six plants were inoculated with each construct in at least three independent experiments. At 5 days post inoculation (dpi), mild mosaic symptom appeared on the control PVX-inoculated plants. The plants inoculated with PVX-V1, PVX-C2, and PVX-C3 produced PVX-like symptoms without any other discernible phenotype through continuous observation (Figure S2). However, obvious mosaic and downward curling of newly emerging leaves were observed in the PVX-V2-infected plants at 5 dpi. Furthermore, the upper leaves became crinkled and necrotic lesions appeared on the inoculated leaves at 9 dpi, which was followed by apical necrosis at 13 dpi that ultimately led to the death of the plants (Figure 4a). To determine whether severe symptoms are the consequence of higher virus accumulation, Northern blotting was employed to examine the RNA accumulation of PVX-V2. There was no obvious difference in the accumulation of PVX genomic RNA (gRNA) between PVX-V2-infected plants and PVX-infected plants at 5 dpi and 9 dpi, while the accumulation of triple gene block subgenomic RNA (TGB sgRNA) and CP sgRNA were apparently upregulated at 9 dpi in PVX-V2-infected plants compared to PVX-infected plants (Figure 4b), which suggests that V2 can enhance the expression of TGB sgRNA and CP sgRNA. These results indicate that V2 is a virulence determinant inducing severe crinkling and necrosis when expressed from a PVX-based vector.

Plants inoculated with PVX-C1 showed mild leaf curling at 5 dpi, which was followed by strong downward curling and crinkling of the newly emerging leaves. The inoculated and systematic leaves, but not the apical leaves, developed visible necrotic lesions at 13 dpi, while the plants inoculated with PVX did not show any necrosis (Figure 4a). The cell death during pathogen infection is a typical feature of necrotic tissue. Therefore, the production of H_2_O_2_ in PVX-C1-infected leaves was investigated by in situ detection with the DAB uptake method. In the presence of H_2_O_2_, DAB can polymerize to produce deep brown products which can be visualized after decolorization by ethanol. Figure 4e shows that H_2_O_2_ accumulated to a higher level in the systemically PVX-C1-infected leaves, especially at the base, at 14 dpi. In contrast, there was no obvious accumulation of H_2_O_2_ in PVX-infected leaves. Northern blotting was conducted to quantify viral RNA accumulation, showing that the gRNA was not affected in the presence of C1, while the TGB sgRNA and CP sgRNA increased substantially at 9 dpi and 13 dpi in PVX-C1-infected plants compared to PVX-infected plants (Figure 4c). Taken together, these results show that AGV C1 enhances the pathogenicity of PVX eliciting necrotic lesions in *N. benthamiana*.

The inoculation of PVX-C4 on *N. benthamiana* induced upward leaf curling at 5 dpi. The symptoms aggravated progressively with time. Hyperplasia and strong leaf upward curling could be observed in the upper leaves at 9 dpi and 13 dpi (Figure 4a). Northern blotting showed that there were no differences in viral gRNA or sgRNA accumulation between PVX-C4- and PVX-infected plants (Figure 4d), which revealed that C4 does not change the replication and expression of PVX. However, AGV C4 can induce hyperplasia and severe upward leaf curling in *N. benthamiana*, which implies that C4 could be a putative symptom determinant.

### 3.4. The N-terminal Acylated Sites of C4 are Associated with its Function as Symptom Determinant

**Figure 5:**
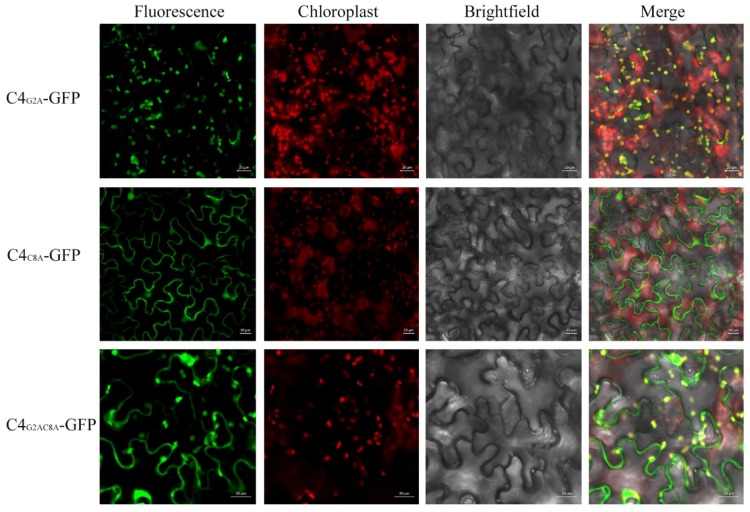
Subcellular localization of AGV C4 mutant variants on N-terminal acylated sites in *N. benthamiana* leaves. The recombinant fluorescent protein C4_G2A_-GFP expressed from pCam35S-C4_G2A_-GFP is mainly localized to chloroplasts, C4_C8A_-GFP expressed from pCam35S-C4_C8A_-GFP is targeted to plasma membrane, and nucleus and C4_G2AC8A_-GFP expressed from pCam35S-C4_G2AC8A_-GFP is localized in the cytoplasm, nucleus, and chloroplasts. The chloroplasts are identified by the red auto-fluorescence of chlorophyll. Confocal laser scanning images were taken approximately 3 d post infiltration. Scale bars indicated in the photos represent 20 μm.

In view of the fact that AGV C4 is a putative symptom determinant, the key amino acids involved in establishing its subcellular localization or symptom determinant function were further investigated through several point mutations. N-myristoylation is a common modification in some C4 proteins of geminiviruses, so we predicted the potential post-translational modifications of AGV C4 with several online programs (http://lipid.biocuckoo.org/, http://lishuyan.lzu.edu.cn/seqpalm/, http://bioinfo.ncu.edu.cn/WAP-Palm.aspx). The results showed that the Gly residue at position 2 and Cys residue at position 8 were predicted to be acylated by N-myristoylation and S-palmitoylation, respectively. The residues Gly2 and Cys8 were substituted with Ala, independently and simultaneously, resulting in C4 mutants C4_G2A_, C4_C8A_, and C4_G2AC8A_. To identify whether the putative acylated modifications affect the localization of C4, we fused the C4 mutants with GFP obtaining C4_G2A_-GFP, C4_C8A_-GFP, and C4_G2AC8A_-GFP. Figure 5 shows that the green fluorescence of C4_G2A_-GFP mainly colocalized with the red auto-fluorescence of chloroplasts with low fluorescence in the cytoplasm, plasma membrane, and nucleus, which indicates that C4_G2A_-GFP is mainly targeted to chloroplasts. In contrast, there was no chloroplast localization in the C4_C8A_-GFP. The fluorescence of C4_G2AC8A_-GFP was detected in the cytoplasm, plasma membrane, nucleus, and chloroplasts, similar to that of C4-GFP (Figure 5 and Figure S1).

**Figure 6:**
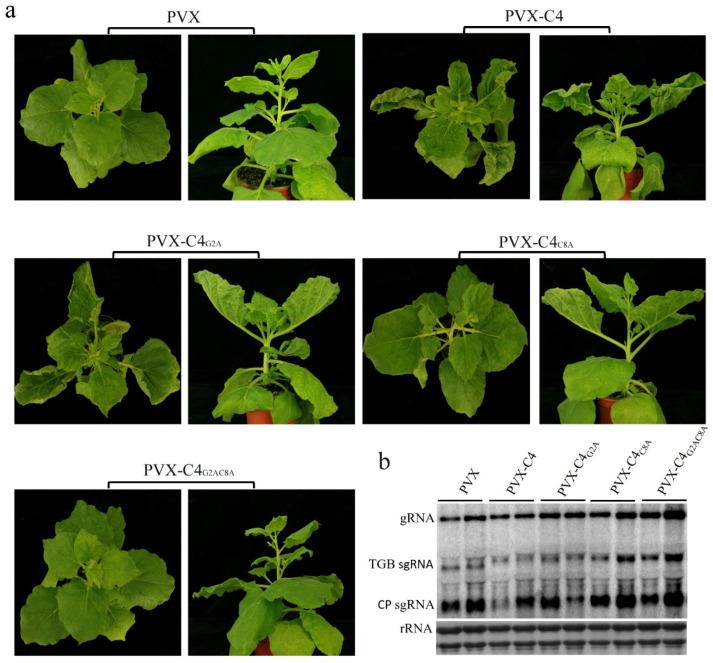
The N-terminal acylated residues of C4 are associated with its symptom determinant function. (**a**) Symptoms of *N. benthamiana* plants inoculated with PVX, PVX-C4, and PVX-C4 mutant variants mutated in its N-terminal potentially acylated sites. Symptoms were photographed at 21 dpi. The images shown are representative plants out of six plants inoculated with each construct in at least three independent experiments. PVX-C4_G2A_ showed PVX-C4-like phenotype with severe upward leaf curling, PVX-C4_C8A_ induced very mild upward leaf curling, and PVX-C4_G2AC8A_-infected plants showed PVX-like symptoms. (**b**) Northern blotting analyses of viral RNA accumulation showed that there were no obvious differences among PVX-C4 mutant variants-inoculated plants compared to PVX- and PVX-C4-inoculated plants. The blot shown is a representative one out of three independent experiments. The probe used was specific for PVX RNA.

Furthermore, to investigate the effect of AGV C4 mutants on symptom formation, the PVX vector was used to express the C4 mutant variants. Six plants for each construct were infected in at least three independent experiments. At the early stage of infection (5 dpi), the plants infected with PVX-C4 and its derivatives developed mild upward leaf curling. At the late stage of infection (21 dpi), the plants infected with PVX-C4_G2A_ showed PVX-C4-like phenotypes with severe upward leaf curling. However, PVX-C4_C8A_ induced very mild upward leaf curling and the PVX-C4_G2AC8A_-infected plants showed no differences in phenotype compared with plants inoculated with PVX (Figure 6a). In order to eliminate the effect of virus accumulation on symptom development, Northern blotting was conducted to analyze the viral RNA accumulation of PVX-C4, PVX-C4_G2A_, PVX-C4_C8A_, and PVX-C4_G2AC8A_. The results demonstrate that there were no differences in viral gRNA and sgRNA accumulation among PVX-C4- and C4 mutant variants-infected plants (Figure 6b). Taken together, our results show that the residues G2 and C8 are essential for C4 localization and play important roles in AGV C4 function as a putative symptom determinant.

## 4. Discussion

Viruses are obligate intracellular pathogens with relatively small genomes encoding very few proteins, which means that they depend heavily on host factors to facilitate infection and counter host defense responses [15]. The subcellular localization of viral proteins provides clues to understand the interaction with host factors and their functions during virus infection. The AGV C1 is the replication-associated protein (Rep) with conserved motifs with the Rep protein of other geminiviruses [12]. The cytoplasmic and nuclear localization of AGV C1 is in agreement with its function as Rep. The C2 and C3 proteins of monopartite geminiviruses function as transcriptional activator and replication enhancer, respectively [16]. The localization of AGV C2 and C3 to the nucleus implies that they are possibly involved in the transcription and replication of the AGV genome. The chloroplast localization suggests that AGV C4 may perform some different functions than other C4 homologs. V1 is the coat protein (CP) of monopartite geminiviruses and it exerts multiple functions, including mediating vector transmission, shuttling of genome DNA into and out of the nucleus, and facilitating cell-to-cell or systemic viral movement [16]. The AGV V1 displays the same localization than CPs of many monopartite or bipartite geminiviruses, such as tomato yellow leaf curl virus (TYLCV) [17] or tomato leaf curl Java virus [18]. The cytoplasmic and nuclear localization of AGV V2 is in agreement with its function as RSS of PTGS.

It is widely accepted that the virus-derived small-interfering RNAs (vsiRNAs)-mediated RNA silencing pathway is an important plant immune mechanism during virus infection [19]. In this antiviral defense pathway, vsiRNAs are produced from viral dsRNA precursors by a set of Dicer-like proteins, and then assembled with Argonaute proteins to form the RNA-induced silencing complex. In this complex, the vsiRNAs are used to guide specific cleavage of the cognate viral RNAs. This process is referred to as PTGS, which is a ubiquitous defense mechanism against RNA and DNA viruses. Inhibition of RNA silencing by virus-encoded RSS proteins is an essential strategy adopted by viruses in the arms race between plants and viruses. In geminiviruses, several proteins (i.e. AC2/C2, AC4/C4, V2, and βC1) have been identified as RSSs targeting different steps of the PTGS pathway [20,21,22,23,24]. For example, the V2 protein of TYLCV was identified to suppress PTGS through its interaction with SGS3 to prevent it from accessing substrate RNA [25,26]. SGS3 is a dsRNA-binding protein which recognizes the 5´ overhang dsRNA for subsequent steps in PTGS [27]. In this study, we identified AGV V2 as a RSS targeting PTGS using the co-agroinfiltration assays with 35S-GFP in 16c plants. It cannot be excluded that there are other RSSs in AGV. Many RNA silencing suppressors have also been identified as determinants of pathogenicity in plants. We have also found that AGV V2 expressed from a PVX-based vector produced serious mosaic, crinkling and necrosis symptoms in *N. benthamiana*. The hypersensitive response-like (HR-like) response was not observed when we expressed AGV V2 by infiltration of agrobacterium cultures harboring the vector pCHF3-V2 under the control of cauliflower mosaic virus 35S promoter (Figure 3a), which indicates that AGV V2 does not behave like a HR elicitor in *N. benthamiana*. Therefore, we speculate that the severe symptoms of PVX-V2-infected *N. benthamiana* plants were induced by the enhanced accumulation of PVX TGB sgRNA and CP sgRNA, which may be attributed to the activity of V2 as an RSS.

Expression of C1 from the PVX-C1 construct induced crinkling and necrosis symptoms in *N. benthamiana*, and H_2_O_2_ accumulation can be detected in the systemically infected leaves. However, there was no HR-like response in the leaves transiently expressing C1 protein by agroinfiltration from the 35S promoter (Figure 3a). These results suggest that AGV C1 is not a HR elicitor in *N. benthamiana*, but it potentially enhances the pathogenicity of PVX by increasing the accumulation of PVX TGB sgRNA and CP sgRNA.

The C4 protein is the least conserved of all geminivirus proteins, and it has a divergent biological function [16]. Many C4 proteins from different geminiviruses alter the host plant development and have been proved to be symptom determinants [16,28]. For example, transgenic *Arabidopsis* plants expressing beet curly top virus (BCTV) and beet severe curly top virus (BSCTV) C4 exhibit symptoms similar to those cause by the viral infection, which are induced by ectopic cell division [29,30]; the C4 protein of sweet potato leaf curl virus (SPLCV) alters plant development by regulating brassinosteroid signaling through the interaction with AtBIN2 [31]; and tomato leaf curl Yunnan virus (TLCYnV) C4 induces cell division through enhancing the stability of Cyclin D 1.1, which regulates the G1/S-phase transition in plants [32]. We have proved that AGV C4 can induce severe upward leaf curling in *N. benthamiana* by expressing it from a PVX-based vector. In addition, PVX-C4 did not change the PVX gRNA and sgRNA accumulation, which implies that C4 can induce the symptom formation as a symptom determinant.

Acylation is a post-translational modification that has been reported in several geminivirus C4 proteins. For example, the plasma membrane localization of BCTV C4 is dependent on its intact N-terminal myristoylation motif [29], and the mutation of the N-myristoylation site disrupted its plasma membrane localization and the induction of hyperplasia [33]. In addition, S-palmitoylation also occurs in some geminivirus C4 proteins [34]. For example, the palmitoylation of African cassava mosaic Zanzibar virus (EACMZV) AC4 contributed to its membrane localization [34]. In our research, the subcellular localization studies showed that AGV C4 protein exhibits a complex localization with strong fluorescent signal in the cytoplasm, plasma membrane, nucleus, and chloroplasts. The mutation of the putative N-myristoylation site in C4 (C4_G2A_) reduced its localization at the plasma membrane and nucleus, so that this mutant version was mainly targeted at chloroplasts. Unlike the BCTV C4, the AGV C4_G2A_ mutant still induced PVX-C4-like symptoms. The mutation of the putative S-palmitoylation site in C4 (C4_C8A_) abolished its localization in chloroplasts. Furthermore, C4_C8A_ displayed a reduced induction of upward leaf curling at the late stage of infection. The simultaneous mutant on the N-myristoylation and the S-palmitoylation sites (C4_G2AC8A_) displayed a similar localization to that of C4, while induced PVX-like symptom at the late stage of the virus infection. We speculate that there are no obvious connections between localization and pathogenicity of AGV C4. In view of the C4 localization, the putative S-palmitoylation modification is important for its chloroplast localization; the putative N-myristoylation modification is important for its plasma membrane localization; competitive balance may exist between the N-myristoylation and the S-palmitoylation of C4. In consideration of pathogenicity, N-myristoylation and, especially, S-palmitoylation, play essential roles in the function of C4 as a symptom determinant.

Taking into account all results obtained in this study, we conclude that: (I) AGV V2 is a suppressor of PTGS; (II) AGV V2 and C1 enhance the pathogenicity of PVX inducing severe crinkling and necrosis; and (III) AGV C4 is a putative symptom determinant and acylation is important for its localization and symptom induction.

## Supplementary Materials

The following are available online at http://www.mdpi.com/1999-4915/10/9/488/s1, Figure S1: Subcellular fractionation of C4-GFP, C4_G2A_-GFP, C4_C8A_-GFP and C4_G2AC8A_-GFP. Figure S2: The symptoms of PVX-V1-, PVX-C2- and PVX-C3-inoculated *N. benthamiana* plants show PVX-like symptoms. Table S1: Primers used for PCR amplification and probe synthesis.
