# Supplementary material for: Functional Scanning of Apple Geminivirus Proteins as Symptom Determinants and Suppressors of Posttranscriptional Gene Silencing
Source: Viruses. 2018 Sep 11;10(9):488. doi: 10.3390/v10090488 (PMC6164617; doi:10.3390/v10090488)
Supplement: Supplementary file 1 [file viruses-10-00488-s001.zip › Table S1.docx]

**T**able S1 Primers used for PCR amplification and probe synthesis.

| Name | Sequence | Enzyme |
| --- | --- | --- |
| PVX/pCHF-V1-F1 | GGATCCATCGATatggcctcgatggggagaaagag | BamHI ClaI |
| PVX/pCHF-V1-R1 | GCGGCCGCTCTAGAttaatgatagtaaatcattcgag | NotI XbaI |
| PVX/pCHF-V2-F1 | GGATCCATCGATatggaaaccgtgttctctc | BamHI ClaI |
| PVX/pCHF-V2-R1 | GTCGACtcaggatatagagatgatcctg | SalI |
| PVX/pCHF-C1-F1 | GGTACCATCGATatgccacgagagcccaacac | KpnI ClaI |
| PVX/pCHF-C1-R1 | GCGGCCGCTCTAGAttaaggggagcttgtggtggggt | NotI XbaI |
| PVX/pCHF-C2-F1 | GGATCCATCGATatgccAtctttAtctttctctcc | BamHI ClaI |
| PVX/pCHF-C2-R1 | GTCGACctacttctcaaagaggacgct | SalI |
| PVX/pCHF-C3-F1 | GGATCCATCGATatggattctcgcacaggg | BamHI ClaI |
| PVX/pCHF-C3-R1 | GTCGACttaataaagaagcatttttacatCag | SalI |
| PVX/pCHF-C4-F1 | GGATCCATCGATatggggagcctcatctccac | BamHI ClaI |
| PVX/pCHF-C4-R1 | GCGGCCGCTCTAGActagatgctggccctgcccc | NotI XbaI |
| cam-gfp-V1-5F | ATACCCGGGGAATGGACTCGAGGGGGAGA | SmaI |
| cam-gfp-V1-3R | AGCTCTAGAATGGAAATATATCATTCGAG | XbaI |
| cam-gfp-V2-5F | ATACCCGGGGAATGGAAGCCGTGTTC | SmaI |
| cam-gfp-V2-3R | AGCTCTAGAGGATATAGAGATGATCCTGTT | XbaI |
| cam-gfp-C1-5F | ATACCCGGGGAATGCCACGAGAGCCCAA | SmaI |
| cam-gfp-C1-3R | AGCTCTAGAAGGGGAGCTTGTGGTG | XbaI |
| cam-gfp-C2-5F | ATACCCGGGGAATGCCGTCTTTGTCTTTCTC | SmaI |
| cam-gfp-C2-3R | AGCTCTAGACTTCTCAAAGAGGACGCTG | XbaI |
| cam-gfp-C3-5F | ATACCCGGGGAATGGATTCTCGCACAGG | SmaI |
| cam-gfp-C3-3R | AGCTCTAGAATAAAGAAGCATTTTTACATCAG | XbaI |
| cam-gfp-C4-5F | ATACCCGGGGAATGGGGAGCCTCATCTC | SmaI |
| cam-gfp-C4-3R | AGCTCTAGAAATGCTGGCCCTGCCC | XbaI |
| PVX-C4_G2A_-5F | AGCATCGATATGGCGAGCCTCATCTC | ClaI |
| PVX-C4_C8A_-5F | AGCATCGATATGGGGAGCCTCATCTCCACGGCCTTA | ClaI |
| PVX-C4_G2AC8A_-5F | AGCATCGATATGGCGAGCCTCATCTCCACGGCCTTA | ClaI |
| cam-gfp-C4_G2A_-5F | ATACCCGGGGAATGGCGAGCCTCATCTC | SmaI |
| cam-gfp-C4_C8A_-5F | ATACCCGGGGAATGGGGAGCCTCATCTCCACGGCCTTA | SmaI |
| cam-gfp-C4_G2AC8A_-5F | ATACCCGGGGAATGGCGAGCCTCATCTCCACGGCCTTA | SmaI |
| PVX-Probe-5F | CACAACACAGCCCATAGGGTC |  |
| PVX-Probe-3R | TTAACATCCAGTTCCATACCACTG |  |
