# Supplementary figures and images for: Functional Scanning of Apple Geminivirus Proteins as Symptom Determinants and Suppressors of Posttranscriptional Gene Silencing
Source: Viruses. 2018 Sep 11;10(9):488. doi: 10.3390/v10090488 (PMC6164617; doi:10.3390/v10090488)

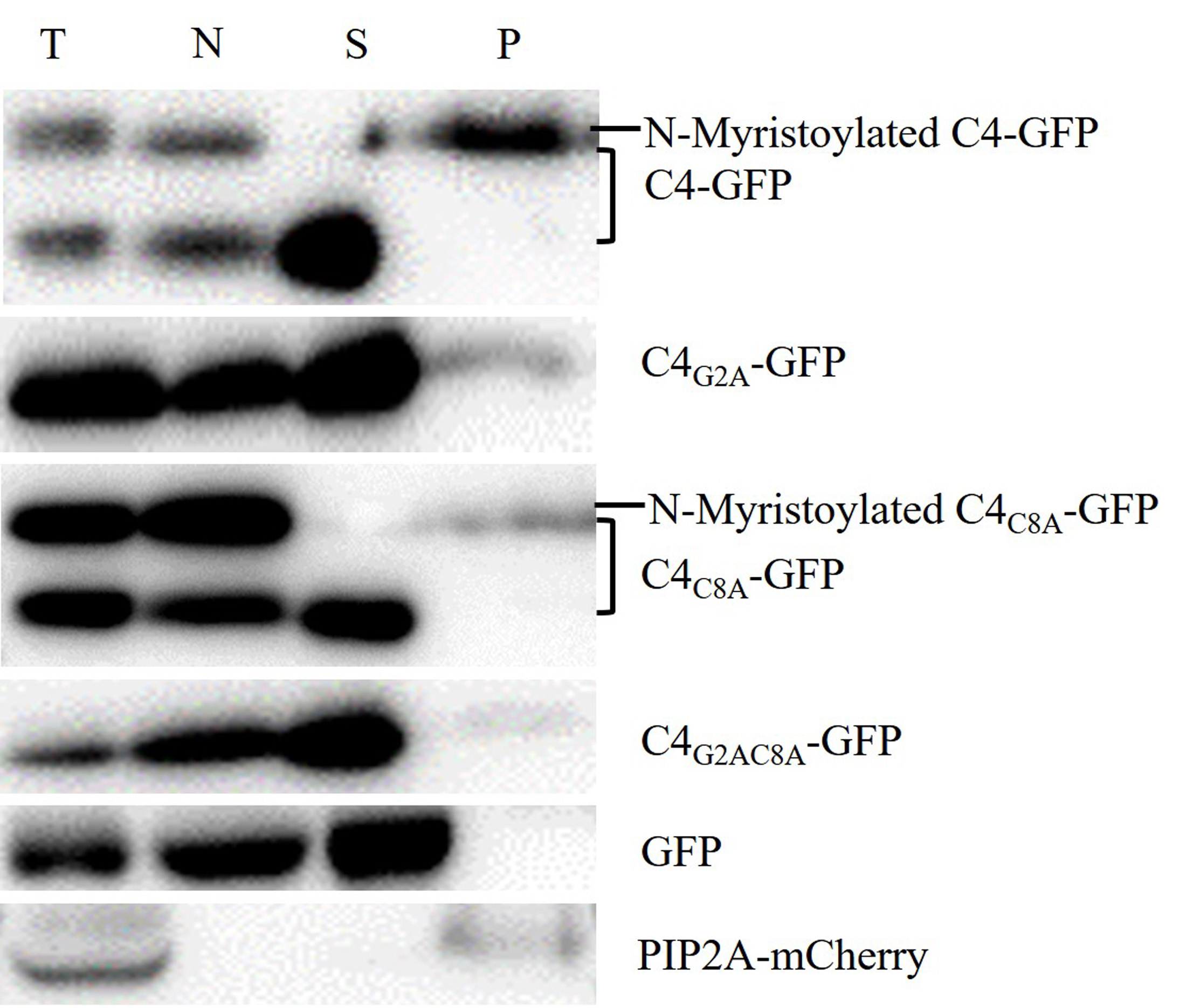

Supplement: Supplementary file 1 [file viruses-10-00488-s001.zip › Figure S1.jpg]

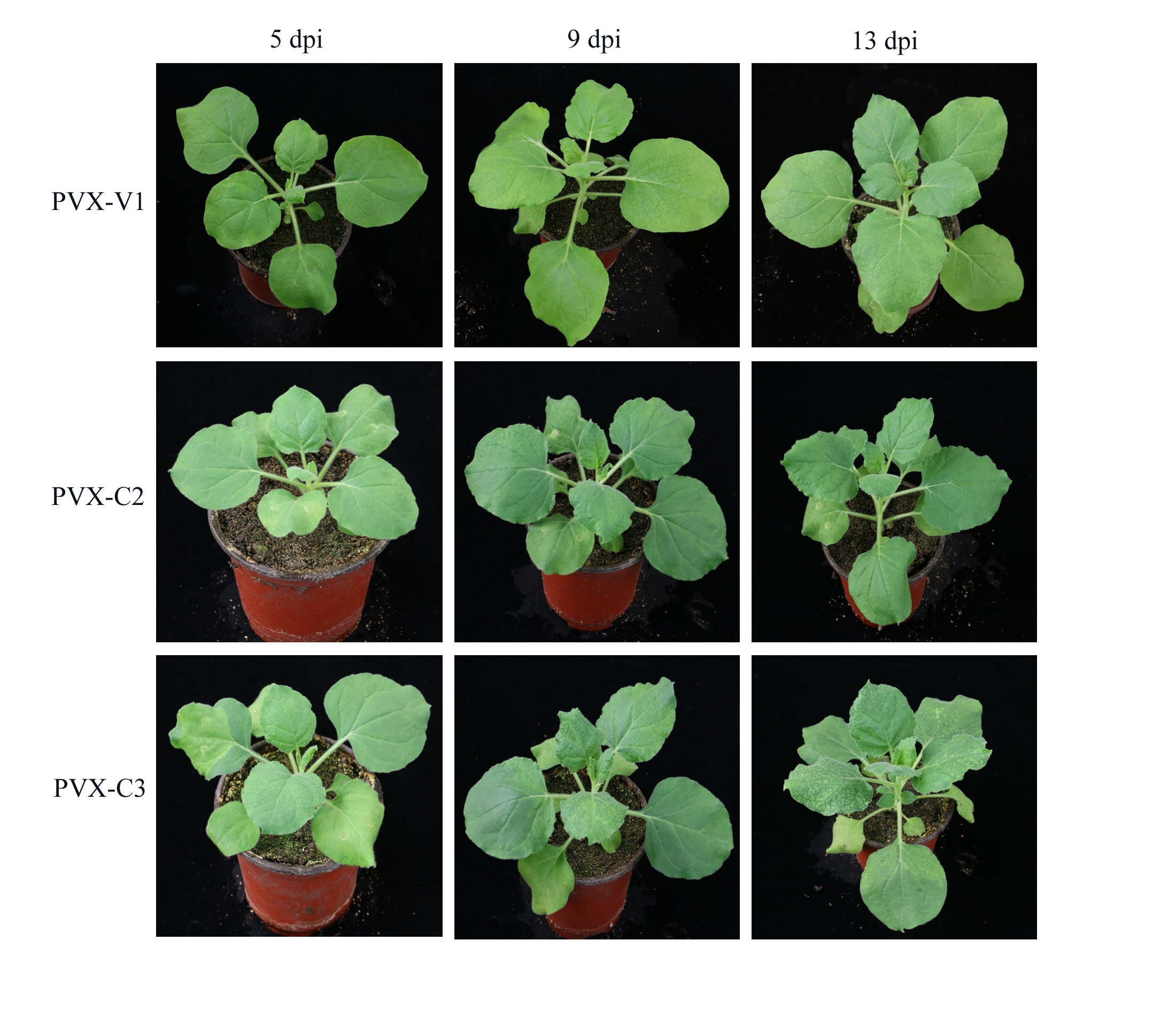

Supplement: Supplementary file 1 [file viruses-10-00488-s001.zip › Figure S2.tif]
